# Mental states and personality based on real-time physical activity and facial expression recognition

**DOI:** 10.3389/fpsyt.2022.1019043

**Published:** 2023-01-09

**Authors:** Yating Huang, Dengyue Zhai, Jingze Song, Xuanheng Rao, Xiao Sun, Jin Tang

**Affiliations:** ^1^School of Mental Health and Psychological Sciences, Anhui Medical University, Hefei, China; ^2^Hefei Comprehensive National Science Center, Institute of Artificial Intelligence, Hefei, China; ^3^Department of Neurology, The Third Affiliated Hospital of Anhui Medical University, Hefei, China; ^4^ZhongJuYuan Intelligent Technology Co., Ltd., Hefei, China

**Keywords:** emotion calculation, expression recognition, five factor model, Russell's circumplex model, mental health

## Abstract

**Introduction:**

To explore a quick and non-invasive way to measure individual psychological states, this study developed interview-based scales, and multi-modal information was collected from 172 participants.

**Methods:**

We developed the Interview Psychological Symptom Inventory (IPSI) which eventually retained 53 items with nine main factors. All of them performed well in terms of reliability and validity. We used optimized convolutional neural networks and original detection algorithms for the recognition of individual facial expressions and physical activity based on Russell's circumplex model and the five factor model.

**Results:**

We found that there was a significant correlation between the developed scale and the participants' scores on each factor in the Symptom Checklist-90 (SCL-90) and Big Five Inventory (BFI-2) [*r* = (−0.257, 0.632), *p* < 0.01]. Among the multi-modal data, the arousal of facial expressions was significantly correlated with the interval of validity (*p* < 0.01), valence was significantly correlated with IPSI and SCL-90, and physical activity was significantly correlated with gender, age, and factors of the scales.

**Discussion:**

Our research demonstrates that mental health can be monitored and assessed remotely by collecting and analyzing multimodal data from individuals captured by digital tools.

## 1. Introduction

Affective computing is an interdisciplinary study involving multiple fields including computer science, cognitive science, and psychology (1). In the field of psychology, an individual's internal emotional experience can be inferred based on the individual's external facial expressions, gesture expressions, and intonation expressions (2). With the concept of affective computing proposed in 1997, the characteristic physiological and behavioral signals caused by human emotions are obtained through various sensors to establish an “emotional model” (3), so as to accurately identify human emotions (4) and eliminate uncertainties and ambiguities. Therefore, the computation of emotions and feelings is no longer limited to traditional methods such as autonomic nervous system measurements, startle response measurements, and brain measurements. Current methods of measuring emotion combined with deep learning are less effective in general environments (5). Different measures also make it difficult to keep the results of emotion research consistent. However, methods proposed in the field of human recognition can be used more effectively (6) in psychological research and are suitable for real-time monitoring of the mental state of healthy people in a universal environment.

Analysis of facial expressions is crucial in psychological analysis (7, 8). To calculate facial emotions, psychological theory, mainly based on the circumplex model of emotions was proposed by Russell in 1980 (9), which considers the division of continuous emotions as a ring in a two-dimensional plane composed of valence and arousal. The implementation of recognition points in a two-dimensional coordinate system to express individual emotions, thus recognizing static and continuous facial expressions (10) allows for more rapid acquisition of real-time emotional data of individuals and recognizing emotions (11). The validity and reliability of the facial prediction model of the Symptom Checklist-90 (SCL-90) were confirmed by studies showing that mental health can be identified from faces (11). A model based on the Five Factor Model (FFM) revealed a link between objective facial image cues and general personality traits (12), and the risk of depression expressed through personality is also captured by FFM (13). The possibility of using neural networks trained on large labeled datasets to predict multidimensional personality profiles from face morphological information has been demonstrated (5). Pound et al. (14) found that facial symmetry can predict individual extroversion. The facial width-to-height ratio is related to individual characteristics such as achievement striving, deceit, dominance, and aggression. Body movements have been identified as a manifestation of many abnormal psychological states (15). Studies have shown that people with mild behavioral impairment have different behavioral manifestations related to the neurodegeneration of mental illness (16, 17).

Body movement recognition is based on human keypoint detection, a pre-task of human behavior recognition, which aims to accurately locate the position of human joints in the image (18). Wang et al. (19) compared the spatio-temporal, time-domain, and frequency-domain characteristics of gait between patients who are depressed and healthy people in a dynamic video and found differences in joint activity between the two groups in body swaying, left-arm/right-arm swing, and vertical head movement, which is beneficial to further promote depression identification programs.

Finding suitable datasets in multimodal sentiment analysis for behavioral quantification and emotional expression has been a considerable challenge. Some data regarding different modalities were publicly available in previous studies (20), such as the SSPNet Conflict Corpus multimodal sentiment portrait of Geneva. However, most high-quality still image datasets were obtained by having actors perform emotions and then photographing their expressions or tagging the images (21). There was no link has been established between the sources of these multimodal datasets and the traditional psychological measures of psychological states used to scale self-assessment. Open-ended psychological questions are not subject to reliability testing like standard psychological scales and thus are more deficient in practical use.

In this study, we attempted to simulate the use of interviews to acquire uncontrolled multi-modal videos of participants throughout the process of psychological counseling by asking them psychological questions. We analyzed facial expressions and physical activity in conjunction with the results of psychological scales. We hope that a real-time contact-free measurement perspective of an individual's mental health in a non-professional setting might be useful for the identification of mental status and personality. Valuable directions may also be developed for researchers in the field of affective computing. Our study aimed to answer the following: (a) Does the scale developed according to the SCL-90 show good reliability and validity? (b) Is the original algorithm for monitoring physical activity levels valid, and does the physical activity of individuals correlate with their mental state? (c) Are participants' facial expressions and physical activity levels related to their mental state or personality?

## 2. Materials and methods

### 2.1. Participants

A total of 200 participants ranging from 12 to 77 years and of any occupation were recruited in August and September 2021 in Hefei, Anhui Province, China. All participants provided informed consent and could withdraw from the study at any time. In the case of participants under the age of 18 years, their parents provided informed consent. The participants first completed the SCL-90 and the Big Five Inventory (BFI-2) before participating in multimodal data collection for interviews. To mitigate the effects of having too many questions and repeated measures, participants were interviewed within 3 h of completing the questionnaires. Ultimately, 172 participants were included in this study. There were 81 male participants and 91 female participant, with M*age* = 45.77 years and SD*age* = 25.81 years. Among the participants, 23% were 12–18 years old, 45% were 19–60 years old, and 31% were over 60 years old. We excluded (a) Participants with minimal or maximal scores on the SCL-90 and BFI-2, (b) Participants with diagnosed psychiatric disorders, (c) Participants with more than one scale item missing or video data less than 10,800 frames, and (d) Participants with diseases that may affect the experiment, such as facial palsy and Parkinson's.

### 2.2. Instruments

#### 2.2.1. SCL-90

The SCL-90 (22), developed by Derogatis, provides a simple way to obtain a series of quantitative indicators to comprehensively describe an individual's mental health. The Chinese version is also used as an identification indicator (23, 24). We focused on the scores of the main factors of the scale to evaluate the mental health status of the participants. In this study, we obtained the reliability of the scale Cronbach's α = 0.973 and the validity of Kaiser–Meyer–Olkin (KMO) = 0.887.

#### 2.2.2. BFI-2

We used the Chinese version of the BFI-2 (25) based on FFM personality theory, which showed good reliability and validity across multiple groups in different countries. In this study, we obtained α = 0.714 and KMO = 0.786.

#### 2.2.3. Interview question development-IPSI

Based on the SCL-90 scale, we changed the way each old question was asked to create a more colloquial pool of items containing a total of 90 items from the original SCL-90 scale, providing more optionality for subsequent item selection. The interview questionnaire was designed to reveal the rich inner activity of the participants. Each item was set up as a question for the participant to answer regarding whether or not and when the participant made an initial choice. Then, an open-ended question was created after each situation to guide further responses detailing their condition or similar symptoms they had experienced in the past. One point was scored when the participant experienced the symptoms in the project in the last 2 weeks. The camera recorded the entire test, and no additional recording was required for the main test other than the score.

All interviewers were experienced counselors or therapists who were trained to participate. After a small pilot survey, the Interview Psychological Symptom Inventory (IPSI) was revised by two psychologists. The experts evaluated the items mainly by judging whether they accurately expressed the content to be measured in the dimension, eliminating items that did not match the interpretation of the dimension, revising sentences that were ambiguous, illogical, or abstract in the description, and considering the interview length of the items. After deletions and modifications, 57 scale items were finally determined. A total of 20 members of the general public were then invited to correct the fluency and accuracy of the questionnaire in order to make the project more understandable to the participants.

### 2.3. Procedure

**Figure 1:**
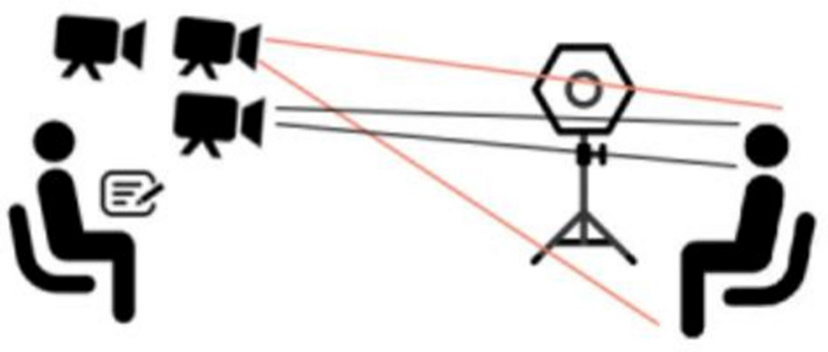
Seating and equipment placement of interviewers and participants in rooms where the video was captured. The third camera was used to prevent interruptions in the process of saving the video to the cloud in real time.

The interviews were conducted in three quiet rooms with the same scene arrangement, as shown in Figure 1. To separately capture the frontal face and full body video of the participants, each room was placed with three 1,080-pixel cameras of different heights, and fill-in panels were used when necessary. The interviewer and the participant sat face to face. The cameras were placed about 1.6 m from the ground, focusing on the head and the whole body of the participants. Recordings were made with a frame rate of 25 Hz, capturing an image resolution of up to 3,264 × 2,448 with an automatic focus of 5–50 mm. We controlled the distance between the participant's seat and the interviewer to be >1.5 m. This was done to eliminate the effect of distance on the intensity of facial movements and to ensure that the cameras captured an unobstructed frontal full-body shot of the participants. After obtaining basic information regarding the participants, the interviewers asked questions in the IPSI in a sequence that lasted 35 to 50 min. All personal information was kept completely secret.

The consultation scenario was simulated to collect data, in order to keep the stimuli consistent. We preferred to analyze the expressions and physical activity of the participants in non-medical scenarios.

### 2.4. Statistical analysis

#### 2.4.1. Facial expression recognition

The single-frame face image from the participant's video was preprocessed and cropped to 256 × 256 after facial recognition. We performed 2D convolution on the three-channel RGB face image and the output feature map size was unchanged and kept at 64 channels. The normalization function InstanceNorm and the activation function ReLU were used for further processing, and the feature map size remained unchanged. We used multiple 3 × 3 2D convolutional layers to process the feature map. Residual connections were used between layers, and the feature size was 128 × 128 × 256.

**Figure 2:**
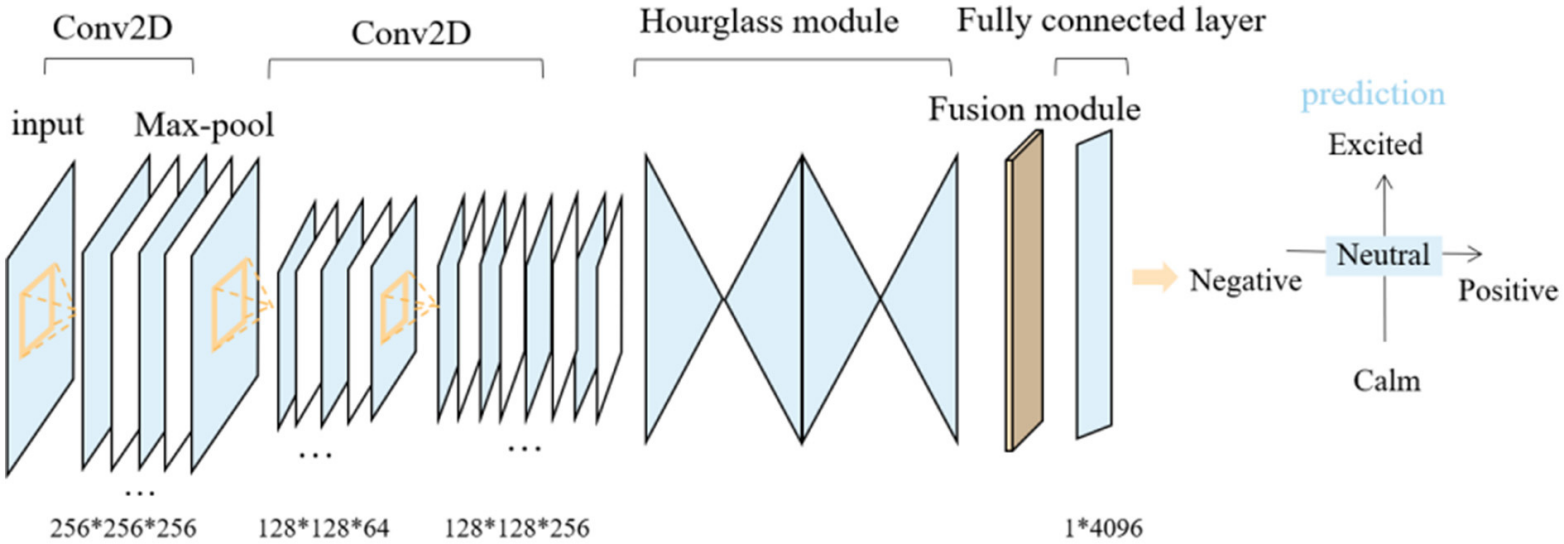
Recognition of facial expression, arousal and valence. The publication of the information in this figure was agreed to and authorized by the participant.

We obtained the key point area of the face for face feature correction. Two cascaded fourth-order Hourglass networks were used to pre-train the face at 68 key points. The feature information at multiple scales is fused by downsampling, upsampling and residual modules. Facial expression recognition requires consideration of minute local features. A 2D convolutional neural network, avg pooling, max pooling, and dimensionality reduction were used sequentially for feature fusion to output 1 × 4,096 feature vectors. The predicted values of output arousal and validity through the fully connected layer were normalized to the mathematical space of [−1, 1]. The vertical axis is the arousal intensity of the emotion, with higher scores indicating a stronger physiological or psychological response to external stimuli. The horizontal axis −1 indicates the most negative emotional potency and the closer to 1, the more positive the emotion. The facial expression recognition process is shown in Figure 2.

In this study, we proposed a loss function to reduce the absolute error in the regression:


(1) $$\begin{array}{l} \left(\text{Y},\hat{\text{Y}}\right)=\frac{\alpha }{\alpha +\beta }{\text{L}}_{\text{MAE}}\left(\text{Y},\hat{\text{Y}}\right)+\frac{\beta }{\alpha +\beta }{\text{L}}_{\text{PCC}}\left(\text{Y},\hat{\text{Y}}\right) \end{array}$$

Where α and β are random values between 0 and 1, and they are not 0 at the same time. ${\text{L}}_{\text{MAE}}\left(\text{Y},\hat{\text{Y}}\right)$ represents the mean absolute error:


(2) $$\begin{array}{l} {\text{L}}_{\text{MAE}}\left(\text{Y},\hat{\text{Y}}\right)={\text{M}A\text{E}}_{\text{valence}}\left(\text{Y},\hat{\text{Y}}\right)+{\text{M}A\text{E}}_{\text{arousal}}\left(\text{Y},\hat{\text{Y}}\right) \end{array}$$

(3) $$\begin{array}{l} \mathrm{MAE}\left(\text{Y},\hat{\text{Y}}\right)=\frac{1}{\text{n}}\overset{\text{n}}{\underset{\text{i}=1}{\sum }}|{\text{Y}}_{\text{i}}-{\hat{\text{Y}}}_{\text{i}}| \end{array}$$

Where y_*i*_ and ${\hat{\text{y}}}_{i}$, respectively, denote the predicted and labeled value of arousal and valence corresponding to face image *i*, and ${\text{L}}_{\text{PCC}}\left(\text{Y},\hat{\text{Y}}\right)$ represents the Pearson correlation coefficient. Its loss function is as follows:


(4) $$\begin{array}{l} {\text{L}}_{\text{PCC}}\left(\text{Y},\hat{\text{Y}}\right)=1-\frac{{\text{P}C\text{C}}_{\text{valence}}\left(\text{Y},\hat{\text{Y}}\right)+{\text{P}C\text{C}}_{\text{arousal}}\left(\text{Y},\hat{\text{Y}}\right)}{2} \end{array}$$

(5) $$\begin{array}{l} \text{P}C\text{C}\left(\text{Y},\hat{\text{Y}}\right)=\frac{\text{E}\left(\text{Y}-\mu_{\text{Y}}\right)\left(\hat{\text{Y}}-\mu_{\text{Y}}\right)}{\sigma_{\text{Y}}\sigma_{\text{Y}}} \end{array}$$

$\mu_{\text{Y}},\mu_{\hat{\text{Y}}}$ represent the expected value, and $\sigma_{\text{Y}},\sigma_{\hat{\text{Y}}}$ represent the standard deviation.

We used two datasets for training. AffectNet is an open-source large-scale image dataset annotated with arousal and valence. It contains 420,000 images annotated by sentiment experts. However, the dataset has few images of Asians. The average per-class accuracy of model performance on the AffectNet is 0.70 (26) [AffectNet baseline = 0.58 (27)]. In order to achieve high accuracy for training on Asian faces, the second dataset consisted of pictures from the internet collected by crawlers, comprising about 10,000 pictures of Asian faces. We annotated this dataset using semi-automatic annotation. An annotator was trained to annotate the data using the existing standard dataset of arousal and validity. The confidence levels below 75% were then manually checked and rescaled. Transfer learning significantly improves the accuracy of the model for Asian faces.

#### 2.4.2. Physical activity

**Figure 3:**
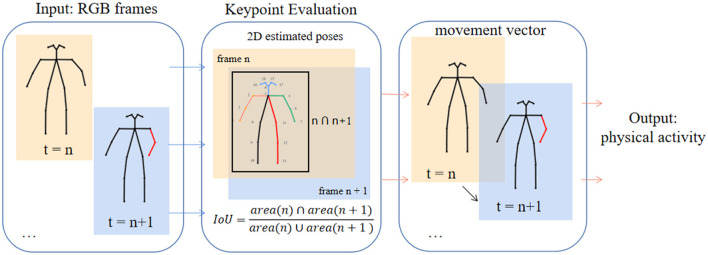
The process of calculating the physical activity of the participant in every two frames of the image. The publication of the information in this figure was agreed to and authorized by the participant.

We characterized and extracted motion information based on the identification and detection of 18 key points of the participants in each frame of the video using OpenPose. Each participant's bounding box was tracked by the intersection-over-union (IoU) algorithm to track the overlap between the candidate bound and the ground truth bound to exclude other characters that may appear in the video. The sequence of key points where the target person appears in the video was obtained with five positions of the head, hand, and leg as the vectors to be measured. The differences between the five vectors of adjacent frames were calculated, and the mean value of the mode length was taken. Taking the mean value can avoid some errors due to undetectable body parts. We normalized the values between 0 and 1 according to the bounding box, where higher values indicate higher body activity. As shown in Figure 3, a physical activity value can be output between the two frames.

The participants themselves and the two experts rated the participants' physical activity on a five-point scale, rated as follows: very active, more active, average, relatively inactive, and very inactive. The two participants that the experts agreed as the most and least active were used as references. In this way, the videos of all participants were scored at the end of the interview for all participants. The scores of the two experts for one participant were taken as the average value.

## 3. Results

**Figure 4:**
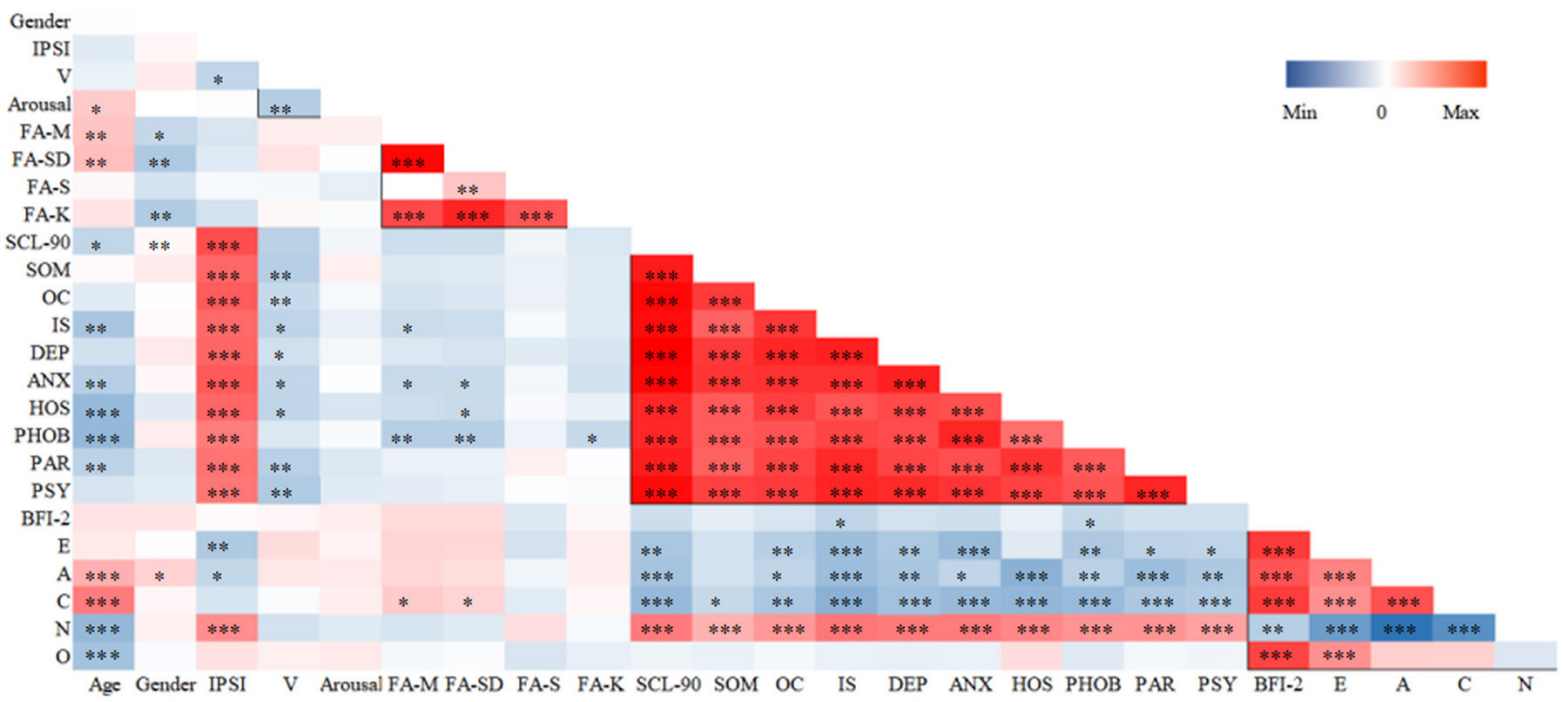
Relationship between participants' psychological scale scores and multimodal data. *p < 0.05, **p < 0.01, ***p < 0.001. IPSI, Interview Psychological Symptom Inventory; V, valence; PA-M, the mean of Physical Activity; PA-SD, the standard deviation of Physical Activity; PA-S, the skewness of Physical Activity; PA-K, the kurtosis of Physical Activity; SOM, Somatization; OC, Obsessive-Compulsive; IS, Interpersonal- Sensitivity; DEP, Depression; ANX, Anxiety; HOS, Hostility; PHOB, Phobic Anxiety; PAR, Paranoid Ideation; PSY, Psychoticism; E, Extraversion; A, Agreeableness; C, Conscientiousness; N, Neuroticism; O, Openness.

**SCL-90 BFI-2** participant scale scores were processed using SPSS 22.0. In this study, participants' SCL-90 score was *M* = 131.83 and *SD* = 37.50 and the BFI-2 score was *M* = 17 and *SD* = 1. The scores for the subscales in the SCL-90 and the five dimensions in the BFI-2 are detailed in Table 1. Correlations between intra-scale, inter-scale, and multi-modal data were analyzed. The highest correlations were found for each factor within the SCL-90 ∈ [0.516, 0.910] (*p* < 0.01), and significant correlations were found between factors within the BFI-2 ∈ [−0.623, 0.700] except for openness, which was not significantly correlated with agreeableness, conscientiousness, and neuroticism. Some of the BFI-2 scores were significantly correlated with the SCL-90 scale factors [−0.424, 0.484]. The relationships between the scales are shown in Figure 4, and detailed correlation data are presented in the Supplementary material.

**Table 1:** Scores on the SCl-90 and BFI-2 scales and subscales.

	**Min**	**Max**	***M***	***SD***
SCL-90	90	330	131.83	37.50
SOM	1	3.42	1.35	0.43
O-C	1	3.80	1.78	0.55
I-S	1	3.67	1.54	0.55
DEP	1	4	1.52	0.52
ANX	1	3.50	1.41	0.48
HOS	1	4	1.38	0.50
PHOB	1	3.57	1.35	0.52
PAR	1	3.67	1.37	0.46
PSY	1	3.60	1.33	0.41
BFI-2	13	20	17	1
E	21	54	3.30	0.47
A	34	60	4.03	0.42
C	28	60	3.85	0.57
N	16	49	2.46	0.55
O	23	57	3.32	0.5

SOM, Somatization; OC, Obsessive-Compulsive; I-S, Interpersonal-Sensitivity; DEP, Depression; ANX, Anxiety; HOS, Hostility; PHOB, Phobic Anxiety; PAR, Paranoid Ideation; PSY, psychoticism; E, Extraversion; A, Agreeableness; C, Conscientiousness; N, Neuroticism; O, Openness.

The Interview Psychological Symptom Inventory items 5, 7, 18, 19, and 54 were found to be less correlated with the scale score according to statistical analysis with SPSS 22.0, the reliability was improved from α = 0.895 to α = 0.898 after deletion. IPSI with split-half reliability α = 0.854, KMO = 0.890, and Barrett's spherical *p* = 0.000. Exploratory factor analysis results contained nine factors with factor loadings ∈ [0.554–0.784] and accumulation = 96.929%. The adapted interview-style document ultimately retained 53 items. The **M**_*IPSI*_ = 11.713, **SD**_*IPSI*_ = 8.267, and the threshold score of 20 for the correlation between the participants' scores on the interview scale and the SCL-90 score was used to identify a mental state with suspected symptoms. The items included in each factor are shown in Table 2, and all factors are significantly correlated, *p* < 0.001 (Supplementary Table 3). The content of the original IPSI is detailed in the Supplementary material.

**Table 2:** Number of items and factor loadings included in the IPSI subscale.

	**Number of items**	**Item numbers**	**Factor loading**
SOM	5	C1, C12, C40, C49, C52	0.554
OC	5	C45, C46, C51, C55, C65	0.712
I-S	5	C6, C36, C37, C41, C61	0.707
DEP	7	C14, C15, C20, C26, C29, C32, C54, C79	0.736
ANX	8	C2, C23, C39, C57, C72, C78, C80, C86	0.784
HOS	6	C11, C24, C63, C67, C74, C81	0.689
PHOB	3	C50, C70, C82	0.558
PAR	4	C8, C43, C76, C83	0.687
PSY	5	C16, C62, C84, C85, C88	0.647
others	4	C44, C59, C64, C89	0.725

SOM, Somatization; OC, Obsessive-Compulsive; I-S, Interpersonal-Sensitivity; DEP, Depression; ANX, Anxiety; HOS, Hostility; PHOB, Phobic Anxiety; PAR, Paranoid Ideation; PSY, psychoticism.

### 3.1. Multi-modal data

The mean values of arousal and valence for all participants were (0.24, −0.16). K-means clustering was used to quickly classify participants' arousal and valence, resulting in two categories of values, with final cluster centers of 0.23 and 0.25 for arousal and 0.04 and −0.26 for valence (Supplementary Figure 5). In the two groups of participants with SCL-90 scores < 160 and ≥ 160 (*n* = 142, *n* = 30), the values of arousal and valence were statistically significant according to a *t*-test and the Mann–Whitney U-test (*p* < 0.05). The time-series data of participants' physical activity were processed into four values: mean, standard deviation, skewness, and kurtosis. After dimensionality reduction, the extraction of the two common factors explained 94.82% of the variance. The highest correlation (α = 0.97) between physical activity skewness and other common factors was obtained by the maximum variance method. All data from participants were averaged to represent each participant's physical activity level, and matched in parallel with participants' self-reports and expert labels, with matching rates of 91.86 and 84.30%, respectively, and a correlation coefficient of 0.873, which demonstrates the good empirical validity of our method.

## 4. Discussion

Statistical results between the scales showed that the SCL-90 subscale is negatively correlated with extraversion (E), agreeableness (A), and conscientiousness (C), with positive correlations for neuroticism (N) in FFM. In the sub-dimensions, respectfulness ∈ [−0.424, −0.174] (*p* < 0.05), organization ∈ [−0.303, −0.175] (*p* < 0.05), anxiety ∈ [0.226, 0.372] (*p* < 0.01), depression ∈ [0.277, 0.531] (*p* < 0.01), and emotional volatility ∈ [0.160, 0.392] (*p* < 0.05) were correlated with all sub-dimensions of SCL-90. In this study, the positive correlation of hostility (HOS) and N were the most significant (*p* < 0.01), and HOS was also negatively correlated with age, continuum valence changes in facial expressions, and A and C in personality. This finding is similar to a study that found that borderline and schizotypal patients are more hostile and aggressive (28), and a study that showed that psychological symptoms correlate moderately with personality disorders (29). SCL-90 subscale scores may be negatively correlated with studies examining personality disorders with altered functional connectivity with precuneus (30), but the current findings are insufficient to constitute other inferences.

The Interview Psychological Symptom Inventory builds on the SCL-90s “well-defined questions” by changing the way the questions are asked and hiding conclusive information so that the questions after “yes/no” do not have a clear direction. The open-ended scale is designed to capture the external manifestations of emotions that participants may evoke in non-neutral stimulus situations. It is the first attempt to adapt widely used reliability and validity. It facilitates access to participants' multimodal information rather than recommending a stimulus to elicit a single emotion. The small number of participants did not reach five times the number of items, otherwise, the data might have been analyzed more accurately.

This study extracted identifiable facial expressions and physical activity, as well as multimodal information on psychological scales. We found significant correlations between the validity of facial expressions and somatization (SOM), obsessive-compulsive (OC), interpersonal-sensitivity (IS), anxiety (ANX), HOS, paranoid ideation (PAR), and psychoticism (PSY), while none of the correlations between arousal and individual mental states and personality traits were significant. The results of our study provide a basis for the expression of facial emotions and individual mental states. In the treatment of psychologically related disorders, the separation of facial expressions into different emotional dimensions may allow for the study of impaired facial emotional expressions in patients with mental illness (31). Furthermore, the facial action coding system (FACS) (32) is a method that can objectively quantify pain-related facial expressions in patients with Alzheimer's.

However, there is no relevant publicly available dataset with human annotation for a consistent comparison of physical activity recognition. We tried to reanalyze the four features by principal component analysis and by weighting them into one dataset, but the results were not significantly different. Extending body posture to gait recognition and monitoring when the characteristics of limb movements are significantly different in psychiatric disorders vs. healthy controls can predict patients who are depressed with high accuracy (33–35). Differential emotion expression between mental disorders and healthy populations (36) has been demonstrated. Furthermore, the mechanisms associated with complex neural networks that regulate and control the gestural expression of complex emotions have not been fully investigated (37). In future research, it may be possible to extract the exact site of visualized individual emotional expression by decomposing features of the facial coding system (3) or body skeleton.

Overall, our results show that subtle changes in spontaneous facial expressions and limb movements do correlate with some mental states and personality traits. The feasibility of being able to use multimodal monitoring results as a basis for the detection of mental states will increase as the accuracy of the model improves. Identifying the mental states of individuals in everyday life is highly scalable. The methodology and findings of the contactless assay could be useful for future deployment in sites, such as schools, to achieve low-cost intelligent experiments that could complement traditional psychological assessment methods where speed is more important than accuracy and the ethical implications of the use of related technologies require rigorous auditing.

## 5. Conclusion

In this study, we used an innovative method based on the SCL-90 to develop an interview-based semi-open psychological scale to capture participants' psychological traits and videos and to analyze their facial expressions and physical activity. Each individual also completed the SCL-90 and BFI-2 for a multimodal study. Significant correlations were found between mental status and selected subscales of the FFM. The participants' facial expressions and physical activity were also significantly correlated with their mental states and personality traits, which provide a strong basis for the relationship between individual behavioral performance and psychology.

## Data availability statement

The raw data supporting the conclusions of this article will be made available by the authors, without undue reservation.

## Ethics statement

The studies involving human participants were reviewed and approved by Ethics Committee of Anhui Medical University. Written informed consent to participate in this study was provided by the participants' legal guardian/next of kin.

## Author contributions

YH and DZ: conceptualization and writing—review and editing. YH, XR, and JS: methodology. XR and JS: software. JS: validation. YH: formal analysis, investigation, data curation, writing—original draft, and visualization. DZ: resources. XS: supervision. JT: project administration. JT and XS: funding acquisition. All authors have approved of the version of the manuscript to be submitted.
