# Supplementary material for: Mental states and personality based on real-time physical activity and facial expression recognition
Source: Front Psychiatry. 2023 Jan 9;13:1019043. doi: 10.3389/fpsyt.2022.1019043 (PMC9868243; doi:10.3389/fpsyt.2022.1019043)
Supplement: Supplementary file 1 [file Table_1.DOCX]

**Supplementary Material**

Table1 Participants' scores on SCL-90 and BFI-2.

|  | Min | Max | Mean | SD |
| --- | --- | --- | --- | --- |
| SCL-90 | 90 | 330 | 131.83 | 37.50 |
| SOM | 1 | 3.42 | 1.35 | 0.43 |
| O-C | 1 | 3.8 | 1.78 | 0.55 |
| I-S | 1 | 3.67 | 1.54 | 0.55 |
| DEP | 1 | 4 | 1.52 | 0.52 |
| ANX | 1 | 3.5 | 1.41 | 0.48 |
| HOS | 1 | 4 | 1.38 | 0.50 |
| PHOB | 1 | 3.57 | 1.37 | 0.52 |
| PAR | 1 | 3.67 | 1.37 | 0.46 |
| PSY | 1 | 3.6 | 1.33 | 0.41 |
| BFI-2 | 161 | 240 | 203.42 | 14.36 |
| E | 21 | 54 | 39.59 | 5.61 |
| A | 34 | 60 | 48.35 | 5.06 |
| C | 28 | 60 | 46.15 | 6.85 |
| N | 16 | 49 | 29.49 | 6.56 |
| O | 23 | 57 | 39.83 | 6.00 |
| Sociability | 5 | 19 | 12.92 | 2.86 |
| Assertiveness | 4 | 18 | 12.37 | 2.25 |
| Energy | 8 | 20 | 14.3 | 2.48 |
| Compassion | 12 | 20 | 16.49 | 1.94 |
| Respectfulness | 9 | 20 | 15.92 | 2.21 |
| Trust | 8 | 20 | 15.95 | 2.34 |
| Organization | 8 | 20 | 14.99 | 2.97 |
| Productiveness | 8 | 20 | 15.37 | 2.74 |
| Responsibility | 7 | 20 | 15.79 | 2.43 |
| Anxiety | 4 | 18 | 11.05 | 2.48 |
| Depression | 4 | 17 | 8.98 | 2.79 |
| Emotional volatility | 4 | 19 | 9.46 | 2.94 |
| Intellectual curiosity | 7 | 19 | 13.5 | 2.07 |
| Aesthetic sensitivity | 4 | 20 | 12.48 | 3.05 |
| Creative imagination | 5 | 20 | 13.85 | 2.83 |
| age | 10 | 77 | 45.77 | 21.81 |
| gender | 0 | 1 | 0.53 | 0.50 |

Table 2 Correlation between each subscale. *NOTE.* *P < 0.05, **P < 0.01.

| gender | 0.004 |  |  |  |  |  |  |  |  |
| --- | --- | --- | --- | --- | --- | --- | --- | --- | --- |
| IPSI | -0.089 | 0.029 |  |  |  |  |  |  |  |
| valence | -0.062 | 0.075 | -.183* |  |  |  |  |  |  |
| arousal | .187* | 0 | 0.013 | -.221** |  |  |  |  |  |
| FA-M | .212** | -.172* | -0.116 | 0.064 | 0.063 |  |  |  |  |
| FA-SD | .235** | -.241** | -0.091 | 0.099 | -0.003 | .883** |  |  |  |
| FA-K | 0.027 | -0.127 | -0.021 | -0.027 | -0.07 | 0.001 | .204** |  |  |
| FA-S | 0.095 | -.230** | -0.129 | 0.026 | -0.013 | .646** | .778** | .606** |  |
| SCL-90 | -.181* | 0.025 | .632** | -.205** | -0.037 | -0.147 | -0.145 | -0.045 | -0.106 |
| SOM | 0.016 | 0.073 | .540** | -.214** | 0.058 | -0.099 | -0.093 | -0.06 | -0.088 |
| OC | -0.087 | 0.006 | .583** | -.166* | -0.024 | -0.123 | -0.108 | -0.059 | -0.094 |
| IS | -.257** | 0.023 | .532** | -.192* | -0.067 | -.154* | -0.148 | -0.024 | -0.092 |
| DEP | -0.134 | 0.075 | .549** | -0.137 | -0.035 | -0.104 | -0.122 | -0.087 | -0.115 |
| ANX | -.218** | 0.029 | .591** | -.182* | 0.004 | -.164* | -.162* | -0.036 | -0.132 |
| HOS | -.310** | -0.086 | .546** | -.187* | -0.111 | -0.148 | -.160* | -0.023 | -0.063 |
| PHOB | -.316** | 0.071 | .466** | -0.102 | -0.018 | -.214** | -.215** | -0.05 | -.168* |
| PAR | -.199** | -0.101 | .503** | -.208** | -0.103 | -0.059 | -0.058 | 0.055 | -0.007 |
| PSY | -0.118 | -0.081 | .488** | -.231** | -0.089 | -0.083 | -0.066 | -0.005 | -0.017 |
| BFI2total | 0.098 | 0.094 | 0.017 | 0.035 | 0.065 | 0.131 | 0.124 | -0.099 | 0.026 |
| E | 0.082 | 0.014 | -.237** | 0.122 | 0.044 | 0.147 | 0.145 | -0.128 | 0.069 |
| A | .284** | .161* | -.177* | 0.091 | 0.075 | 0.143 | 0.12 | -0.04 | 0.062 |
| C | .466** | 0.03 | -0.117 | -0.017 | 0.063 | .187* | .152* | -0.084 | 0.032 |
| N | -.314** | 0.052 | .402** | -0.131 | -0.093 | -0.117 | -0.094 | 0.116 | -0.023 |
| O | -.270** | -0.015 | 0.105 | 0.056 | 0.08 | -0.029 | -0.012 | -0.113 | -0.066 |
| Sociability | 0.077 | 0.063 | -0.127 | 0.121 | -0.03 | 0.124 | 0.137 | -0.122 | 0.08 |
| Assertiveness | 0.147 | -0.061 | -.204** | 0.024 | 0.125 | .219** | .197** | -0.044 | 0.14 |
| Energy | -0.036 | 0.015 | -.204** | 0.116 | 0.021 | -0.01 | -0.008 | -0.108 | -0.063 |
| Compassion | .176* | .153* | 0.085 | 0.031 | -0.028 | 0.025 | 0.067 | -0.028 | 0.023 |
| Respectfulness | .174* | 0.103 | -.207** | 0.038 | 0.13 | 0.056 | 0.027 | -0.013 | -0.03 |
| Trust | .304** | 0.124 | -.257** | 0.136 | 0.062 | .236** | .179* | -0.051 | 0.144 |
| Organization | .355** | 0.006 | -0.112 | -0.039 | 0.01 | 0.145 | 0.1 | -0.075 | 0.011 |
| Productiveness | .468** | 0.068 | -0.129 | -0.003 | 0.05 | .220** | .175* | -0.029 | 0.064 |
| Responsibility | .354** | 0 | -0.048 | 0.002 | 0.108 | 0.103 | 0.109 | -0.114 | 0.006 |
| Anxiety | -.151* | 0.058 | .339** | -0.134 | 0.04 | -0.123 | -0.095 | .152* | 0.006 |
| Depression | -.331** | 0.022 | .376** | -0.092 | -0.092 | -.183* | -0.124 | 0.053 | -0.12 |
| Emotional volatility | -.260** | 0.046 | .255** | -0.092 | -.153* | 0.015 | -0.012 | 0.08 | 0.057 |
| Intellectual curiosity | -.213** | -0.007 | 0.078 | 0.094 | 0.096 | -0.023 | 0.008 | 0.063 | 0.035 |
| Aesthetic sensitivity | -0.121 | 0.099 | 0.112 | 0.03 | 0.145 | -0.095 | -0.079 | -.195* | -.167* |
| Creative imagination | -.287** | -0.134 | 0.046 | 0.018 | -0.056 | 0.057 | 0.054 | -0.076 | 0.014 |
|  | age | gender | IPSI | valence | arousal | FA-M | FA-SD | FA-K | FA-S |

| SOM | .815** |  |  |  |  |  |  |  |  |  |
| --- | --- | --- | --- | --- | --- | --- | --- | --- | --- | --- |
| OC | .882** | .702** |  |  |  |  |  |  |  |  |
| IS | .864** | .562** | .713** |  |  |  |  |  |  |  |
| DEP | .910** | .704** | .775** | .797** |  |  |  |  |  |  |
| ANX | .888** | .722** | .732** | .733** | .794** |  |  |  |  |  |
| HOS | .774** | .588** | .688** | .607** | .653** | .628** |  |  |  |  |
| PHOB | .774** | .586** | .613** | .665** | .640** | .775** | .516** |  |  |  |
| PAR | .805** | .563** | .668** | .763** | .666** | .629** | .731** | .582** |  |  |
| PSY | .870** | .680** | .697** | .778** | .760** | .726** | .677** | .616** | .776** |  |
| BFI-2 | -0.146 | -0.076 | -0.114 | -.186* | -0.131 | -0.143 | -0.067 | -.176* | -0.133 | -0.135 |
| E | -.254** | -0.13 | -.212** | -.290** | -.246** | -.305** | -0.094 | -.245** | -.195* | -.190* |
| A | -.264** | -0.126 | -.187* | -.292** | -.255** | -.186* | -.346** | -.204** | -.291** | -.245** |
| C | -.309** | -.170* | -.249** | -.347** | -.277** | -.305** | -.321** | -.303** | -.245** | -.229** |
| N | .468** | .275** | .365** | .484** | .471** | .464** | .425** | .381** | .382** | .335** |
| O | -0.047 | -0.06 | -0.031 | -0.06 | -0.067 | -0.06 | 0.122 | -0.091 | -0.028 | -0.042 |
| Sociability | -0.117 | 0.005 | -0.092 | -.169* | -0.143 | -.180* | 0.016 | -.155* | -0.079 | -0.081 |
| Assertiveness | -.255** | -.185* | -.251** | -.262** | -.230** | -.297** | -0.114 | -.277** | -0.123 | -0.149 |
| Energy | -.208** | -0.132 | -0.145 | -.224** | -.184* | -.213** | -0.129 | -0.124 | -.240** | -.200** |
| Compassion | -0.037 | 0.024 | -0.017 | -0.063 | -0.051 | -0.017 | -0.097 | -0.009 | -0.087 | -0.029 |
| Respectfulness | -.294** | -.174* | -.253** | -.254** | -.255** | -.209** | -.424** | -.219** | -.284** | -.284** |
| Trust | -.263** | -0.127 | -.152* | -.338** | -.268** | -.190* | -.267** | -.228** | -.289** | -.239** |
| Organization | -.281** | -.175* | -.258** | -.303** | -.219** | -.278** | -.297** | -.242** | -.225** | -.220** |
| Productiveness | -.308** | -0.141 | -.250** | -.347** | -.291** | -.296** | -.337** | -.287** | -.263** | -.229** |
| Responsibility | -.182* | -0.107 | -0.106 | -.219** | -.185* | -.187* | -.161* | -.235** | -0.119 | -0.12 |
| Anxiety | .355** | .226** | .304** | .371** | .342** | .372** | .260** | .274** | .270** | .228** |
| Depression | .467** | .277** | .344** | .531** | .484** | .461** | .354** | .404** | .357** | .372** |
| Emotional volatility | .301** | .160* | .231** | .263** | .303** | .284** | .392** | .236** | .286** | .202** |
| Intellectual curiosity | -0.078 | -0.036 | -0.064 | -0.122 | -0.084 | -0.075 | 0.006 | -0.104 | -0.104 | -0.05 |
| Aesthetic sensitivity | -0.003 | -0.043 | 0.023 | -0.002 | -0.029 | -0.008 | 0.102 | -0.018 | -0.018 | -0.032 |
| Creative imagination | -0.038 | -0.055 | -0.042 | -0.035 | -0.05 | -0.063 | 0.144 | -0.096 | 0.036 | -0.019 |
|  | SCL-90 | SOM | OC | IS | DEP | ANX | HOS | PHOB | PAR | PSY |

| E | .700** |  |  |  |  |  |  |  |  |  |  |  |  |  |  |  |  |  |  |  |
| --- | --- | --- | --- | --- | --- | --- | --- | --- | --- | --- | --- | --- | --- | --- | --- | --- | --- | --- | --- | --- |
| A | .609** | .442** |  |  |  |  |  |  |  |  |  |  |  |  |  |  |  |  |  |  |
| C | .695** | .399** | .620** |  |  |  |  |  |  |  |  |  |  |  |  |  |  |  |  |  |
| N | -.214** | -.439** | -.623** | -.495** |  |  |  |  |  |  |  |  |  |  |  |  |  |  |  |  |
| O | .665** | .393** | .175* | .165* | -0.104 |  |  |  |  |  |  |  |  |  |  |  |  |  |  |  |
| Sociability | .462** | .796** | .342** | .202** | -.352** | .228** |  |  |  |  |  |  |  |  |  |  |  |  |  |  |
| Assertiveness | .549** | .709** | .240** | .385** | -.277** | .312** | .379** |  |  |  |  |  |  |  |  |  |  |  |  |  |
| Energy | .553** | .699** | .387** | .321** | -.336** | .344** | .303** | .257** |  |  |  |  |  |  |  |  |  |  |  |  |
| Compassion | .563** | .311** | .726** | .467** | -.318** | .259** | .249** | .195* | .238** |  |  |  |  |  |  |  |  |  |  |  |
| Respectfulness | .458** | .261** | .787** | .530** | -.528** | .161* | 0.137 | 0.139 | .305** | .362** |  |  |  |  |  |  |  |  |  |  |
| Trust | .417** | .451** | .816** | .453** | -.585** | 0.011 | .402** | .226** | .351** | .399** | .458** |  |  |  |  |  |  |  |  |  |
| Organization | .631** | .358** | .487** | .882** | -.364** | .156* | .173* | .352** | .291** | .367** | .450** | .323** |  |  |  |  |  |  |  |  |
| Productiveness | .637** | .425** | .593** | .879** | -.451** | 0.115 | .242** | .362** | .353** | .452** | .467** | .465** | .707** |  |  |  |  |  |  |  |
| Responsibility | .471** | .210** | .487** | .753** | -.443** | 0.145 | 0.086 | .246** | .153* | .360** | .418** | .358** | .469** | .489** |  |  |  |  |  |  |
| Anxiety | -0.113 | -.403** | -.429** | -.272** | .796** | -0.092 | -.388** | -.194* | -.288** | -.186* | -.317** | -.474** | -.179* | -.307** | -.202** |  |  |  |  |  |
| Depression | -.330** | -.556** | -.587** | -.475** | .818** | -0.126 | -.441** | -.410** | -.377** | -.273** | -.464** | -.605** | -.373** | -.420** | -.412** | .544** |  |  |  |  |
| Emotionalvolatility | -0.069 | -0.112 | -.471** | -.424** | .783** | -0.034 | -0.04 | -0.065 | -0.149 | -.293** | -.470** | -.331** | -.309** | -.348** | -.428** | .416** | .418** |  |  |  |
| Intellectualcuriosity | .552** | .354** | .304** | .186* | -.159* | .694** | .212** | .260** | .321** | .306** | .280** | 0.139 | 0.143 | .197** | 0.127 | -0.104 | -.192* | -0.085 |  |  |
| Aestheticsensitivity | .417** | .195* | 0.027 | 0.071 | -0.044 | .760** | 0.103 | .176* | .164* | 0.125 | 0.041 | -0.084 | 0.065 | 0.027 | 0.089 | -0.02 | -0.036 | -0.048 | .275** |  |
| Creativeimagination | .556** | .364** | 0.118 | 0.138 | -0.056 | .794** | .217** | .281** | .317** | .189* | 0.092 | 0.012 | .156* | 0.07 | 0.12 | -0.097 | -0.088 | 0.041 | .444** | .333** |
|  | BFI2total | Extraversion | Agreeableness | Conscientiousness | Neuroticism | Openness | Sociability | Assertiveness | Energy | Compassion | Respectfulness | Trust | Organization | Productiveness | Responsibility | Anxiety | Depression | Emotionalvolatility | Intellectualcuriosity | Aestheticsensitivity |

Table 3 Correlation between each subscale in the IPSI. *NOTE.* *P < 0.05, **P < 0.01.***P < 0.001.

|  | SOM | OC | I-S | DEP | ANX | HOS | PHOB | PAR | PSY | others |
| --- | --- | --- | --- | --- | --- | --- | --- | --- | --- | --- |
| OC | .389*** |  |  |  |  |  |  |  |  |  |
| I-S | .236** | .551*** |  |  |  |  |  |  |  |  |
| DEP | .407*** | .464*** | .461*** |  |  |  |  |  |  |  |
| ANX | .394*** | .443*** | .460*** | .519*** |  |  |  |  |  |  |
| HOS | .345*** | .459*** | .434*** | .468*** | .489*** |  |  |  |  |  |
| PHOB | .275*** | .347*** | .345*** | .298*** | .503*** | .247** |  |  |  |  |
| PAR | .227** | .383*** | .425*** | .434*** | .568*** | .416*** | .297*** |  |  |  |
| PSY | .229** | .303*** | .454*** | .422*** | .415*** | .456*** | .254** | .472*** |  |  |
| others | .401*** | .543*** | .412*** | .494*** | .504*** | .365*** | .353*** | .421*** | .414*** |  |

Figure 5 Clustering results of arousal and validity of participants' facial expressions.


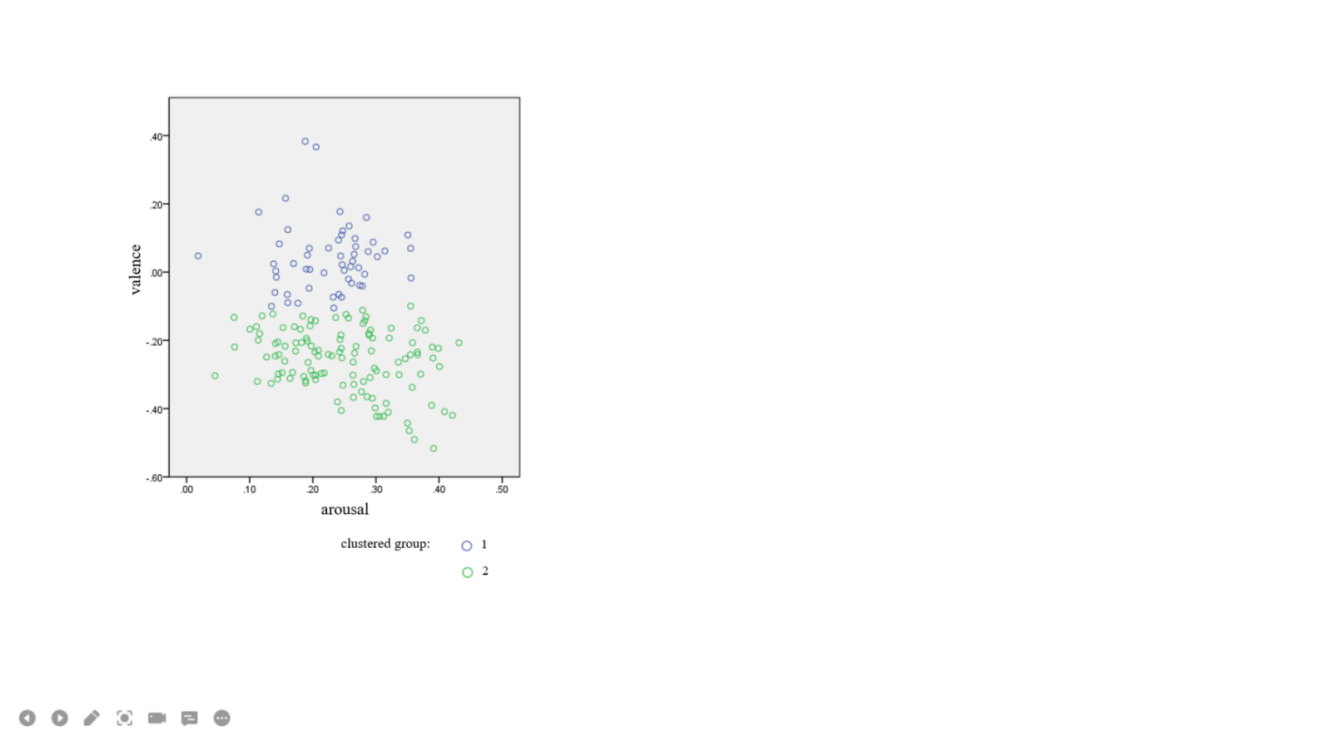


The Interview Psychological Symptom Inventory (IPSI):

**访谈心理症状量表**

使用说明：请您根据访谈问题向来访者提问，并根据来访者的回答选择“是”或“否”，若参来访者回答“是”，您可以根据设定问句进一步提问以便了解来访者的情况。

指导语：在以下的提问中，请您仔细听每一条问题，根据自己最近一周的实际情况作答。当您出现了问题中的症状时，请您尽可能详细的描述自己的情况。

1. 请问您最近是否有头痛的症状？ 是/否

如果若回答是：请问您头痛持续多久了？这种症状是从什么时候开始的？

1. 您最近是否总觉得自己心里不踏实，神经过敏或十分敏感？ 是/否

如果回答是：一般遇到什么事情会觉得自己有些敏感呢？

1. 是否感觉自己对别人责备求全，有些苛责他人？ 是/否

如果回答是：可以请您举一个例子吗，会苛责他人的什么行为呢？

1. 最近有没有责怪别人，觉得别人制造了麻烦？ 是/否

如果回答是：当自己冷静下来后，还会责怪别人吗？

1. 感觉自己容易烦恼和激动吗？ 是/否

如果回答是：是否容易被很小的事情激怒？

1. 最近是否出现了胸痛或胸闷的症状？ 是/否

如果回答是：您自己感觉出现的频率或严重程度如何？

1. 是否感到自己的精力下降，活动减慢？ 是/否

如果回答是：是否感觉自己的外出活动或娱乐活动减少了？

1. 有没有出现过想结束自己的生命的想法？ 是/否

如果回答是：最近是否有过尝试自杀的行为呢？

1. 会不会听到旁人听不到的声音？ 是/否

如果回答是：可以详细描述一下听见的声音是什么样的吗？频率如何呢？

1. 最近是否胃口不好？ 是/否

如果回答是：食欲不好时，自己会怎么应对呢？

1. 最近是否容易哭泣？ 是/否

如果回答是：是有什么事情让您觉得委屈、压抑吗？

1. 是否会无缘无故地突然感到害怕？ 是/否

如果回答是：害怕时是什么样的感受呢？会觉得紧张、心跳加快吗？

1. 是否会不能控制自己，大发脾气？ 是/否

如果回答是：发脾气时是怎样的呢？

1. 最近是否经常责怪、责备自己？ 是/否

如果回答是：是否怕自己连累他人或有自卑感呢？

1. 您是否感到孤独？ 是/否

如果回答是：最近一周经常有这种感觉吗？

1. 最近是否对事物不感兴趣？ 是/否

如果回答是：是否有一些以前的兴趣爱好，现在不想去做了？

1. 是否会觉得别人不理解你、对你没有同理心？ 是/否

如果回答是：什么情境下会有这种感觉呢？

1. 是否觉得别人对你不友好，不喜欢你？ 是/否

如果回答是：什么情况下会有这种感觉呢？

1. 觉得自己心跳跳得很厉害吗？ 是/否

如果回答是：什么时候会跳的厉害呢？

1. 是否觉得恶心或胃部不舒服？ 是/否

如果回答是：最近饮食是否规律？

1. 是否觉得自己比不上他人？ 是/否

如果回答是：是否觉得自己低人一等或一无是处？

1. 是否难以入睡？ 是/否

如果回答是：每晚躺在床上，是否需要半小时以上才能睡着？最近每晚都是这样吗？

1. 做事是不是必须反复检查？ 是/否

如果回答是：是否觉得自己有强迫症状？

1. 是不是难以做出决定？ 是/否

如果回答是：最近您是否常常犹豫不决、反复纠结？

1. 最近是否会感觉自己一阵阵发冷或发热？ 是/否

如果回答是：什么时候会出现这种感觉？

1. 是否会因为感到害怕而避开某些东西、场合或活动？ 是/否

如果回答是：请您描述一下当时的情况可以吗？

1. 是否感觉自己脑子变空了？ 是/否

如果回答是：什么时候脑子是一片空白的？

1. 是否感觉自己的身体发麻或刺痛？ 是/否

如果回答是：什么时候会出现这种感觉呢？

1. 是否觉得自己的前途没有希望？ 是/否

如果回答是：会对自己的未来发展非常不看好吗？

1. 最近总是不能集中注意力？ 是/否

如果回答是：无法集中注意力时，会有什么样的情绪呢？

1. 是否总是感到紧张或认为自己容易紧张? 是/否

如果回答是：是否觉得紧张情绪比较严重？

1. 最近会不会总是想到关于死亡的事？ 是/否

如果回答是：想到与死亡相关的事情，会出现恐惧情绪或其他消极的想法吗？

1. 当别人看着你或谈论你的时候，你会感到不自在吗？ 是/否

如果回答是：可以描述一下自己不自在的感觉吗？

1. 是否觉得有一些不属于自己的想法出现在脑子里？ 是/否

如果回答是：这些想法是什么样的？

1. 有想打人或伤害他人的冲动吗？ 是/否

如果回答是：最近什么时候出现过这种冲动？

1. 最近是否会醒的很早？ 是/否

如果回答是：早醒之后还能够重新入睡吗？

1. 最近出现了必须反复洗手、点数的情况吗？ 是/否

如果回答是：最近出现这种情况的频率如何？

1. 最近是否出现过想摔坏或破坏东西的想法？ 是/否

如果回答是：什么时候会有这种想法？有过实际行为吗？

1. 在商店或电影院等人多的地方，您是否会感到不自在？ 是/否

如果回答是：是否喜欢一个人呆着，不想和他人接触？

1. 会出现一阵阵恐惧或惊恐？ 是/否

如果回答是：恐惧情绪一般由什么事件触发呢？

1. 最近经常与人争论吗？ 是/否

如果回答是：上次与人争论是什么时候？可以描述一下当时的情境吗？

1. 单独一人时，会神经紧张吗？ 是/否

如果回答是：最近是否觉得自己很难放松下来？

1. 会觉得他人对自己的成绩没有做出恰当的评价呢？ 是/否

如果回答是：是否是因为觉得别人对自己的评价比自己预想的偏低？

1. 最近是否会感到坐立不安、心神不定？ 是/否

如果回答是：是否也会觉得自己心情烦躁，心事很多？

1. 会不会觉得自己没有什么价值？ 是/否

如果回答是：会觉得自己一无是处，处处不如别人吗？

1. 是否会感到熟悉的东西、人、情境，逐渐或突然变的陌生？或变得不像是真的？ 是/否

如果回答是：可以举个例子吗？什么时候会出现这种感觉？

1. 会大叫或摔东西吗？ 是/否

如果回答是：当时是否可以控制自己的行为？

1. 是否会害怕自己在公共场合晕倒？ 是/否

如果回答是：或是害怕自己在公众场引入注目吗？

1. 是否觉得别人想占您的便宜？ 是/否

如果回答是：可以举个例子吗？

1. 会由于一些有关性的想法而感到很苦恼吗？ 是/否

如果回答是：您觉得自己的苦恼程度如何呢，会十分困扰自己吗？

1. 是否认为由于自己的有过错，而应当受到惩罚？ 是/否

如果回答是：会常有自责感吗？

1. 会不会认为自己要很快把事情做完，有急迫感呢？ 是/否

如果回答是：拖延事情只会让自己很着急吗？

1. 会不会感到自己有罪？ 是/否

如果回答是：会出现自责感呢？
